# Haemophilus influenzae Type b Meningitis in Children, Eritrea

**DOI:** 10.3201/eid1001.030132

**Published:** 2004-01

**Authors:** Durgadas G. Naik, Melles Seyoum

**Affiliations:** * Central Health Laboratory, Asmara, Eritrea

**Keywords:** Bacterial meningitis, sub-Saharan Africa, “meningitis belt”

**To the Editor:** Bacterial meningitis is a major cause of death and disability in children worldwide: >1,000,000 cases and 200,000 deaths are estimated to occur each year. Neisseria meningitidis, Haemophilus influenzae type b (Hib), and Streptococcus pneumoniae are major causative agents of bacterial meningitis in children. A region in sub-Saharan Africa, extending from Ethiopia in the east to the Gambia in the west and containing 15 countries with >260 million people, is known as the “meningitis belt” because of its high prevalence of endemic disease with periodic epidemics caused by N. meningitidis. 

Eritrea, a small country with an estimated population of 3.5 million in northeast Africa, is part of the meningitis belt. Eritrea gained independence in 1993 and borders the Red Sea on the east, Djibouti on the southeast, Ethiopia on the south, and Sudan on the north. Asmara, with a population of about 500,000, is the capital city. The estimated infant mortality rate is 73 deaths/1,000 live births.

In 2002, a prospective laboratory-based study was carried out in Asmara to gain insight regarding the distribution of bacterial agents causing bacterial meningitis in children. Starting in January 2002, cerebrospinal fluid (CSF) specimens were collected from every child who had a spinal tap administered at Mekane Hiwet Pediatric Hospital, Asmara. This facility serves as the national reference hospital for pediatric care. Within 1 hour of collection, all CSF specimens were processed at the Department of Microbiology, Central Health Laboratory. This laboratory, located <200 m from the Mekane Hiwet Pediatric Hospital, serves as the national reference health laboratory and is the only facility in Eritrea with the capabilities to perform cultures. Standard methods were used to process all specimens and to isolate and identify bacterial agents from the CSF specimens *(**1**–**3**)*. All CSF specimens were cultured on chocolate agar plates with IsoVitalex supplement (BBL Microbiology Systems, Cockeysville, MD) and tested for bacterial antigens by using Wellcogen latex agglutination kits (Remel, Inc., Dartford, UK). Hib strain ATCC 49247 was used as the control strain. All Hib strains were tested for susceptibility to ampicillin, penicillin, chloramphenicol, gentamicin, and cefotaxime by using the disk diffusion method (BBL); isolates determined to be penicillin-resistant were also tested for β-lactamase by using the Nitrocefin touch sticks (Oxoid, Basingstoke, UK).

From January 1 to December 31, 2002, a total of 81 CSF specimens were collected: 38 (47%) were from patients >1 year of age, 28 (35%) from patients 1 to 2 years of age, and 15 (18%) from patients 2 to 14 years of age. Twelve (15%) of the 81 specimens tested positive; 10 were positive by both culture and latex agglutination test (5 Hib, 2 S. pneumoniae, 1 N. meningitidis, and 2 Enterobacteriaceae), and 2 were positive only by the latex agglutination test (1 Hib and 1 N. meningitidis). The patients’ age and sex and the results of microbiologic tests are presented in the Table. 

**Table:** Results of microbiologic tests of five cases of *Haemophilus influenzae* type b^a^

Age/sex	Latex agglutination test	Antimicrobial susceptibility					β-lactamase
Pen	Amp	Cefot	Chl	Genta
3 months/female	Positive	R	R	S	S	S	Positive
5 months/male	Positive	R	R	S	R	R	Positive
6 months/male	Positive	R	R	S	S	S	Positive
1 year/male	Positive	R	R	S	S	S	Positive
3 years/male	Positive	R	S	S	S	S	Positive

^a^Pen, penicillin; Amp, ampicillin; Cefot, cefotaxime; Chl, chloramphenicol; Genta, gentamicin; S, sensitive; R, resistant.

This study preceded the implementation of the Integrated Disease Surveillance (IDS) in Eritrea (last quarter of 2002) and does not allow for calculation of incidence of Hib disease at the national level. However, implementation of IDS will enable microbiologists to prospectively monitor the incidence of infectious diseases, including meningitis caused by Hib.

In many countries, Hib is still reported as a major cause of bacterial meningitis *(**4**–**9**)*, and while Hib meningitis has a relatively low case-fatality rate in developed countries (3% to 5%), high case-fatality rates (20% to 30%) are common in tropical Africa. Rapid laboratory diagnosis and treatment with appropriate antimicrobial drugs, such as third-generation cephalosporins, are crucial in reducing the risk for severe complications. The decrease in Hib meningitis cases after the introduction of Hib vaccination and the use of vaccine to control Hib meningitis are well documented *(**10**–**12**)*. Additionally, the findings of this study suggest that Hib remains the leading cause of childhood meningitis in this region and lead us to advocate for the introduction of vaccination in Eritrea.
